# Advanced laparoscopic HPB surgery: Experience in Seoul National University Bundang Hospital

**DOI:** 10.1002/ags3.12323

**Published:** 2020-03-25

**Authors:** Nepal Kovid, Ho‐Seong Han, Yoo‐Seok Yoon, Jai Young Cho

**Affiliations:** ^1^ Charak Memorial Hospital Pokhara Nepal; ^2^ Seoul National University Bundang Hospital Gyeonggi‐do Korea

**Keywords:** advanced laparoscopy, hepato‐pancreato‐biliary surgery

## Abstract

The worldwide trend in surgery has moved from open surgery to minimally invasive surgery. Likewise, the application of minimally invasive surgery in the hepato‐pancreato‐biliary (HBP) field is also rapidly expanding. The field of HBP surgery can be divided into liver, pancreas and biliary fields. Minimally invasive liver surgery is recently developed. However, laparoscopic liver resection in difficult areas is challenging. However, with the accumulation of experiences, laparoscopic liver resection for difficult areas is performed more than before. With more propagation, more and more liver resection will be performed by laparoscopic approach. In minimally surgery for the pancreas, distal pancreatectomy has become a well‐recommended procedure in benign and borderline malignancy. There have been several systemic reviews that show advantages of laparoscopic distal pancreatectomy. The reports on laparoscopic pancreaticoduodenectomy (PD) are slowly increasing in spite of technical difficulty, with several systemic reviews showing advantages of the procedure. However, more PD will be performed as robotic‐assisted procedures in the future. The laparoscopic surgery for biliary tract malignancy is still in early stages. The laparoscopic surgery for gallbladder cancer has been contraindicated, although there have been encouraging reports from expert centers. The laparoscopic surgery for Klatskin tumor is still an experimental procedure. Robotic‐assisted procedures for the surgery of cholangiocarcinoma will be the future. Robotic‐assisted surgery for the HBP field is still not well‐developed. However, with the necessity of more precise manipulation like intracorporeal suturing, robotic‐assisted surgery will be used more often in the field of HBP surgery.

## 1 INTRODUCTION

Minimally invasive surgery in the field of hepato‐pancreato‐biliary surgery (HPB) has shown contrasting trajectories in its development. On one hand, since the first laparoscopic cholecystectomy in 1985, it is now a routine surgery performed in all corners of the world. On the other hand, the progress in laparoscopic hepatic, pancreatic, and biliary surgeries has been slow when compared to other organs’ surgery and is currently performed only in highly experienced centers by expert surgeons. But, in the last couple of decades, advancement in modern surgical technology, innovations, energy devices, changing concepts and technical expertise has allowed the propagation of minimally invasive surgery in the HBP field. These improvements have now provided laparoscopic HBP surgeons to challenge the difficulties encountered in this field of surgery and to advance to performing advanced laparoscopic surgery. This paper aims to discuss the status of advanced minimally invasive techniques in HPB surgery through our experience in Seoul National University Bundang Hospital.

### 1.1 Laparoscopic liver surgery

With the initial success of laparoscopic cholecystectomy highlighting the advantages of minimally invasive surgery, the procedure was subsequently adapted to liver surgery with the first laparoscopic liver resections (LLR) reported in the early 1990s for benign lesions. The procedures gradually expanded in the next couple of decades to include resections ranging from left lateral sectionectomy to living donor hepatectomies. This propagation of minimally invasive surgery for LLR is attributed to the development of surgical technology, improved surgical skills and change in concepts for liver resection. The landmark in the continued development of LLR was reached in 2008 during the first consensus meeting on laparoscopic liver surgery held in Louisville, Kentucky.^1^ The meeting acknowledged the safety and efficacy of LLR and described its indications as solitary lesions, <5 cm and located in the antero‐lateral segments. This was also an important milestone as LLR was accepted as a standard surgery, even if it was only for left lateral sectionectomy. The second consensus held in Morioka reviewed the status of LLR in comparison with open surgery and made recommendations for LLR. Minor resections considered for standard and major liver resection, although being performed in highly experienced centers, were still considered in the stage of innovation because of the novelty of the procedure and the risks involved.^2^ Based on the recommendations from the meeting, practical guidelines for performing liver resection focusing on the technical issues of LLR have also been reported.^3^

With the advancement of laparoscopic surgical skills and equipment technologies, the indications of LLR are gradually expanding. As experience in performing more complex procedures grows, procedures which were considered to be relative contraindications, like unfavorable locations in the postero‐superior segments, were more performed than before. And with the accumulation of the evidence to support safety of LLR in major hepatectomies and anatomical resections, the number of reports on advanced LLR is also increasing. For LLR to be accepted as standard in these surgeries, the procedure should have outcomes at least similar or better than that of open surgery. The limitations that have prevented the effective propagation of advanced LLR are technical difficulties in parenchymal dissection, hemostasis in unfavourable locations, and the risk of air embolism; all of which are gradually being overcome with experience.

In regards to resections in the unfavorable locations, our team had the privilege to report a case of total laparoscopic right posterior sectionectomy for hepatocellular carcinoma (HCC) in 2006.^4^ Although right posterior sectionectomy is a standard procedure in open surgery, laparoscopic right posterior sectionectomy is still a very challenging procedure. With the developing of better instrument and sealing devices, laparoscopic right posterior sectionectomy has become more technically feasible than before. When laparoscopic right posterior sectionectomy was compared with open surgery, there was no difference in overall survival between laparoscopy and open surgery.^5^

Resection of posterior superior segment is another unfavorable location as it is one of the most difficult parts on which to performing a laparoscopic procedure. In a study comparing posterior superior to anterior lateral segments, laparoscopic approach for posterior superior segment is not inferior to surgery for anterior lateral segment.^6^ Subsequent reports on comparative analysis by case match analysis between posterior superior segment and anterior lateral segment, there was no difference in overall survival and disease‐free survival.^7^ Even though posterior superior is a more difficult procedure, survival outcome appears to be similar between the two groups.

For the minimally invasive surgery for posterior superior lesion, selective use of intercostal trocars can be useful for good operative view. Furthermore, this intercostal trocar facilitates parenchymal resection and bleeding control when posterior superior segment resection is perfromed.^8^, ^9^ Many laparoscopic surgeons have been using this intercostal/transdiaphragmatic trocar when performing resections in the posterior superior segments without any major complications associated with the procedure.^10^, ^11^, ^12^

Centrally located tumors also pose significant technical challenges due to their difficult location. When performing resection for tumor at central lesion, there is high risk of massive bleeding from injury of adjacent vessels. There are reports on LLR for centrally located tumor, which were very close to the hilum or major hepatic vein and inferior vena cava.^13^ The patients were mostly cases of HCC and the operations performed ranged from right posterior sectionectomy, central bi‐sectionectomy, and left major hepatectomy including four caudate lobe resections. When compared with open surgery, there is no difference in survival between laparoscopic surgery and open surgery. Therefore, even for centrally located tumor, laparoscopic resection can be cautiously applied.

Anatomic liver resection is a very important concept in the management of HCC. HCC is usually associated with underlying poor liver function because of chronic liver disease and cirrhosis. And the resection of only the involved segment is beneficial for the patient as it preserves the volume of the remnant liver. The techniques of anatomical liver resection using the Glissonian pedicle approach have proven to be very useful in laparoscopic surgery. Utilizing these concepts, any types of laparoscopic anatomic liver dissection can be possible depending on tumor locations and remnant liver.^14^ The importance of meticulous dissection during liver resection has been highlighted by the impact of remnant liver ischemia as a prognostic factor for survival. The study comparing two groups with minimal remnant liver ischemia and severe remnant liver ischemia showed a big difference in overall survival and disease‐free survival.^15^ Therefore, it is important to perform precise parenchymal resection to minimize remnant liver ischemia. This technique will increase the safety of the operation and the survival as well. In conclusion, owing to technical development and accumulating experiences, advanced laparoscopic liver resections are being performed more often than before, and the limitations brought about by unfavorable locations may be slowly lifted resulting in anatomic liver resection being performed even more widely in the future.

### 1.2 Laparoscopic pancreatic surgery

The use of laparoscopic pancreatic resection has gradually grown in the last 25 years. Numerous reports comparing laparoscopic vs open distal pancreatectomy showed advantages in for the laparoscopic group.^16^ The conclusions from the International Hepato‐Pancreato Biliary Association International Consensus Meeting on minimally invasive pancreatic resection held in 2016 were published through various papers highlighting its advantages.^17^, ^18^, ^19^, ^20^ This was soon followed by the International Summit on Laparoscopic Pancreatic Resection held in Coimbatore, India, in 2016 which focused on the indications for MIPR, identifying the current problems, standardizing techniques as well as developing protocols.^21^ There has been a general agreement among all surgeons regarding the beneficial role of distal pancreatectomy, with many publications regarding the topic (Table 1) including a difficulty scoring system in laparoscopic distal pancreatectomy.^22^

**Table 1:** Summary of systematic reviews on open vs minimally invasive distal pancreatectomy

Authors	Year	Number of studies	Compared variables		
			MIR > OR	MIR = OR	MIR < OR
Jin et al^32^	2012	15	Blood loss, transfusion, SSI, hospital stay, spleen preservation	Operation time, pancreatic fistula	
Nigri et al^33^	2011	10	Blood loss, early oral intake, hospital stay, complications, SSI, pancreatic fistula	Mortality, reoperative rates	
Sui et al^34^	2012	19	Blood loss, transfusion, SSI, hospital stay, early oral intake	Mortality, oncologic clearance	
Xie et al^35^	2012	9	Operative time, early oral intake, hospital stay, spleen preservation		
Pericleous et al^36^	2012	4	Hospital stay	Morbidity, mortality	Operative time
Nakamura et al^37^	2013	24	Blood loss, transfusion, SSI, hospital stay, morbidity		
Mehrabi et al^38^	2015	29	Blood loss, early oral intake, hospital stay	Morbidity, safety	
Ricci et al^39^	2015	5	Blood loss, hospital stay	Morbidity, pancreatic fistula, reoperation, mortality	Operative time
Gavriilidis et al^40^	2018	7	Blood loss, smaller tumors, hospital stay		
Van Hilst et al^41^	2019	21		Overall survival, R0 resection rate, adjuvant chemotherapy	Lymph node yield

Abbreviations: MIR, minimally invasive resection; OR, open resection; SSI, surgical site infection.

Laparoscopic pancreatoduodenectomy, on the other hand, is a highly demanding procedure and is still being performed by very few surgeons in highly experienced centers. Our initial experience of laparoscopic pancreaticoduodenectomy (PD) was between 2004 and 2006, where we reported eight cases, but, considering the difficulties and post‐operative complications associated with the procedure, the procedure's implementation was restricted.^23^ But in 2012, the publication of many good reports which showed significant advantage in lymph node harvest in laparoscopic procedure when compared to open surgery further enhanced its potential benefits, encouraging surgeons to carry out total laparoscopic PD.^24^ But, considering the technical difficulties associated with the use of long instruments during resection, one way of utilizing the benefits of laparoscopic resection may be laparoscopy‐assisted pancreatoduodenectomy.^25^ This allows for the resection to be performed by laparoscopy and the reconstruction performed by minimal invasion. This may aid in decreasing the morbidity of PD as operative stress can be minimized with minimal manipulation of the organ and minimal exposure out to air. So, until the universal use of laparoscopic PD is established, laparoscopic‐assisted PD may be a stepping stone for further development. Table 2 summarizes the systematic reviews comparing the outcomes of open and laparoscopic PD. With the emerging technology of the surgical robot, the flexibility afforded by the technique may be better for performing the anastomosis which makes it a very promising modality for development for the future.^26^, ^27^, ^28^

**Table 2:** Summary of meta‐analysis of open vs laparoscopic pancreatoduodenectomy

Authors	Year	Number of studies	Compared variables		
			MIR > OR	MIR = OR	MIR < OR
Correa‐Gallego et al^42^	2014	6	Less blood loss, lymph node yield	POPF rate, overall complications, morbidity, reoperations	Operative time, Tumor size
Lei et al^43^	2014	9	Blood loss, LOS, wound infection	POPF rate, complications, mortality, lymph node yield	Operative time
Qin et al^44^	2014	11	Blood loss, wound infection, LOS	Overall complications, POPF rate, lymph node yield, reoperation, mortality	Operative time
Pedziwiatr et al^45^	2017	12	Blood loss, LOS, delayed gastric emptying	Overall complications, morbidity	Operative time
Chen et al^46^	2017	26	Blood loss, transfusion rate, LOS	POPF rate, complications, reoperation, readmission, mortality, lymph node yield	Operative time

Abbreviations: LOS, length of stay; MIR, minimally invasive resection; OR, open resection; POPF post‐operative pancreatic fistula.

### 1.3 Laparoscopic biliary surgery

Regarding biliary surgery, laparoscopic cholecystectomy for benign gallbladder disease has been the landmark procedure in terms of its rapid propagation as a universally accessible and feasible technique performed all over the world. Laparoscopic surgery had been contraindicated for gallbladder cancer in favor of open surgery due to the higher chances of cancer cell dissemination and trocar site metastasis associated with intraoperative bile spillage. In 2010, our report of a prospective study with an intention to treat analysis about laparoscopic approach for suspected early stage gallbladder cancer showed that there was no tumor recurrence or metastasis in the patients with a median follow‐up of 24 months.^29^ Ten years of experience later, in 2014, we analyzed the data again where 83 patients suspected of early stage gallbladder cancer were included in the study with a median follow‐up was 60 months. Five‐year survival was 100% for T1a/b and 90.2% for T2 lesions. We have concluded that a favorable long‐term oncologic outcome has been found, and we can apply laparoscopic approach, including lymph node dissection in early gallbladder cancer.

Regarding intrahepatic cholangiocarcinoma, there have been only a few reports on minimally invasive resection. Three years ago, we reported about LLR for cholangiocarcinoma, and the overall survival was acceptable.^30^ For liver or in HBP surgery, one of the untouched and unsolved areas is minimally invasive surgery for hilar cholangiocarcinoma. We reported about five cases of lap liver resection for hilar cholangiocarcinoma 3 years ago which is a very difficult and challenging operation.^31^ Laparoscopy has some limitation for hilar lesion resection because of the need of bilioenteric reconstruction, which may be better performed using a robot. Ultimately, with gathering evidence, in advanced laparoscopic HPB surgery, robots will play a significant role in the future.

## 2 CONCLUSION

Minimally invasive surgery has showed a lot of promise in the field of hepato‐pancreato‐biliary surgery. With advancing technology, expertise and experience, more and more advanced HPB procedures will be performed by minimally invasive surgery and we expect robotic surgery to play a significant role in the future.

## DISCLOSURE

Conflict of Interest: Authors declare no conflict of interests for this article.

## References

[1] Buell JF , Cherqui D , Geller DA , O’Rourke N , Iannitti D , Dagher I , et al. The international position on laparoscopic liver surgery: the Louisville Statement, 2008. Ann Surg. 2009;250(5):825–30. 19916210 10.1097/sla.0b013e3181b3b2d8

[2] Wakabayashi G , Cherqui D , Geller DA , Buell JF , Kaneko H , Han HS , et al. Recommendations for laparoscopic liver resection: a report from the second international consensus conference held in morioka. Ann Surg. 2015;261(4):619–29. 25742461 10.1097/SLA.0000000000001184

[3] Cho JY , Han HS , Wakabayashi G , Soubrane O , Geller D , O’Rourke N , et al. Practical guidelines for performing laparoscopic liver resection based on the second international laparoscopic liver consensus conference. Surg Oncol. 2018;27(1):A5–A9. 10.1016/j.suronc.2017.12.003 29338984

[4] Yoon Y‐S , Han H‐S , Choi YS , Jang J‐Y , Suh K‐S , Kim S‐W , et al. Total laparoscopic right posterior sectionectomy for hepatocellular carcinoma. J Laparoendosc Adv Surg Tech A. 2006;16(3):274–7. 16796440 10.1089/lap.2006.16.274

[5] Cho JY , Han HS , Yoon YS , Choi Y , Lee W . Outcomes of laparoscopic right posterior sectionectomy in patients with hepatocellular carcinoma in the era of laparoscopic surgery. Surgery. 2015;158(1):135–41. 25799467 10.1016/j.surg.2015.02.007

[6] Cho JY , Han HS , Yoon YS , Shin SH . Feasibility of laparoscopic liver resection for tumors located in the posterosuperior segments of the liver, with a special reference to overcoming current limitations on tumor location. Surgery. 2008;144(1):32–8. 18571582 10.1016/j.surg.2008.03.020

[7] Lee W , Han HS , Yoon YS , Cho JY , Choi YR , Shin HK , et al. Comparison of laparoscopic liver resection for hepatocellular carcinoma located in the posterosuperior segments or anterolateral segments: a case‐matched analysis. Surgery. 2016;160(5):1219–26. 27353634 10.1016/j.surg.2016.05.009

[8] Lee W , Han HS , Yoon YS , Cho JY , Choi Y , Shin HK . Role of intercostal trocars on laparoscopic liver resection for tumors in segments 7 and 8. J Hepatobiliary Pancreat Sci. 2014;21(8):E65–E68. 24841194 10.1002/jhbp.123

[9] Guro H , Cho JY , Han HS , Yoon YS , Choi YR , Jang JS , et al. Laparoscopic liver resection of hepatocellular carcinoma located in segments 7 or 8. Surg Endosc. 2018;32(2):872–8. 28730274 10.1007/s00464-017-5756-x

[10] Ichida H , Ishizawa T , Tanaka M , Terasawa M , Watanabe G , Takeda Y , et al. Using transthoracic trocars for laparoscopic resection of subphrenic hepatic tumors. Surg Endosc. 2017;31(3):1280–6. 27444836 10.1007/s00464-016-5107-3

[11] Hirokawa F , Hayashi M , Asakuma M , Shimizu T , Inoue Y , Uchiyama K . Intercostal trocars enable easier laparoscopic resection of liver tumors in segments 7 and 8. World J Surg. 2017;41(5):1340–6. 28097410 10.1007/s00268-016-3867-5

[12] Chiow AKH , Lewin J , Manoharan B , Cavallucci D , Bryant R , O’Rourke N . Intercostal and transthoracic trocars enable easier laparoscopic resection of dome liver lesions. HPB. 2015;17(4):299–303. 25250870 10.1111/hpb.12336 PMC4368392

[13] Yoon YS , Han HS , Cho JY , Kim JH , Kwon Y . Laparoscopic liver resection for centrally located tumors close to the hilum, major hepatic veins, or inferior vena cava. Surgery. 2013;153(4):502–9. 23257080 10.1016/j.surg.2012.10.004

[14] Choi YR , Han HS , Sultan AM , Yoon YS , Cho JY . Glissonean pedicle approach in laparoscopic anatomical liver resection. Hepatogastroenterology. 2014;61(136):2317–20. 25699374

[15] Cho JY , Han HS , Choi YR , Yoon YS , Kim S , Choi JK , et al. Association of remnant liver ischemia with early recurrence and poor survival after liver resection in patients with hepatocellular carcinoma. JAMA Surg. 2017;152(4):386–92. 28052154 10.1001/jamasurg.2016.5040 PMC5470428

[16] Eom BW , Jang JY , Lee SE , Han HS , Yoon YS , Kim SW . Clinical outcomes compared between laparoscopic and open distal pancreatectomy. Surg Endosc. 2008;22(5):1334–8. 18027035 10.1007/s00464-007-9660-7

[17] Kendrick ML , van Hilst J , Boggi U , de Rooij T , Walsh RM , Zeh HJ , et al. Minimally invasive pancreatoduodenectomy. HPB. 2017;19(3):215–24. 28317658 10.1016/j.hpb.2017.01.023

[18] Montagnini AL , Røsok BI , Asbun HJ , Barkun J , Besselink MG , Boggi U , et al. Standardizing terminology for minimally invasive pancreatic resection. HPB. 2017;19(3):182–9. 28317657 10.1016/j.hpb.2017.01.006

[19] Barkun J , Fisher W , Davidson G , Wakabayashi G , Besselink M , Pitt H , et al. Research considerations in the evaluation of minimally invasive pancreatic resection (MIPR). HPB. 2017;19(3):246–53. 28274661 10.1016/j.hpb.2017.01.005

[20] Conlon KC , de Rooij T , van Hilst J , Abu Hidal M , Fleshman J , Talamonti M , et al. Minimally invasive pancreatic resections: cost and value perspectives. HPB. 2017;19(3):225–33. 28268161 10.1016/j.hpb.2017.01.019

[21] Palanivelu C , Takaori K , Abu Hilal M , Kooby DA , Wakabayashi G , Agarwal A , et al. International summit on laparoscopic pancreatic resection (ISLPR) “Coimbatore summit statements”. Surg Oncol. 2018;27(1):A10–A15. 29371066 10.1016/j.suronc.2017.12.001

[22] Ohtsuka T , Ban D , Nakamura Y , Nagakawa Y , Tanabe M , Gotoh Y , et al. Difficulty scoring system in laparoscopic distal pancreatectomy. J Hepatobiliary Pancreat Sci. 2018;25(11):489–97. 30118575 10.1002/jhbp.578

[23] Yoon Y‐S , Han H‐S , Choi Y , Jang J , Kim S , Park Y . Laparoscopic assisted pylorus‐preserving pancreatoduodenectomy performed for cystic tumor in the head of the pancreas. J Korean Surg Soc. 2006;70(6):486–90.

[24] Asbun HJ , Stauffer JA . Laparoscopic vs open pancreaticoduodenectomy: Overall outcomes and severity of complications using the accordion severity grading system. J Am Coll Surg. 2012;215(6):810–9. 22999327 10.1016/j.jamcollsurg.2012.08.006

[25] Mendoza AS , Han HS , Yoon YS , Cho JY , Choi YR . Laparoscopy‐assisted pancreaticoduodenectomy as minimally invasive surgery for periampullary tumors: a comparison of short‐term clinical outcomes of laparoscopy‐assisted pancreaticoduodenectomy and open pancreaticoduodenectomy. J Hepatobiliary Pancreat Sci. 2015;22(12):819–24. 26455716 10.1002/jhbp.289

[26] Zhang J , Wu WM , You L , Zhao YP . Robotic versus open pancreatectomy: a systematic review and meta‐analysis. Ann Surg Oncol. 2013;20(6):1774–80. 23504140 10.1245/s10434-012-2823-3

[27] Cirocchi R , Partelli S , Trastulli S , Coratti A , Parisi A , Falconi M . A systematic review on robotic pancreaticoduodenectomy. Surg Oncol. 2013;22(4):238–46. 24060451 10.1016/j.suronc.2013.08.003

[28] Nassour I , Wang SC , Porembka MR , Yopp AC , Choti MA , Augustine MM , et al. Robotic versus laparoscopic pancreaticoduodenectomy: a NSQIP analysis. J Gastrointest Surg. 2017;21(11):1784–92. 28819886 10.1007/s11605-017-3543-6 PMC5789456

[29] Cho JY , Han HS , Yoon YS , Ahn KS , Kim YH , Lee KH . Laparoscopic approach for suspected early‐stage gallbladder carcinoma. Arch Surg. 2010;145(2):128–33. 20157079 10.1001/archsurg.2009.261

[30] Uy BJ , Han HS , Yoon YS , Cho JY . Laparoscopic liver resection for intrahepatic cholangiocarcinoma. J Laparoendosc Adv Surg Tech A. 2015;25(4):272–7. 25789408 10.1089/lap.2014.0233

[31] Lee W , Han H‐S , Yoon Y‐S , Cho JY , Choi Y , Shin HK , et al. Laparoscopic resection of hilar cholangiocarcinoma. Ann Surg Treat Res. 2015;89(4):228–32. 26448923 10.4174/astr.2015.89.4.228 PMC4595825

[32] Jin T , Altaf K , Xiong JJ , Huang W , Javed MA , Mai G , et al. A systematic review and meta‐analysis of studies comparing laparoscopic and open distal pancreatectomy. HPB. 2012;14(11):711–24. 23043660 10.1111/j.1477-2574.2012.00531.x PMC3482667

[33] Nigri GR , Rosman AS , Petrucciani N , Fancellu A , Pisano M , Zorcolo L , et al. Metaanalysis of trials comparing minimally invasive and open distal pancreatectomies. Surg Endosc. 2011;25(5):1642–51. 21184115 10.1007/s00464-010-1456-5

[34] Sui CJ , Li B , Yang JM , Wang SJ , Zhou YM . Laparoscopic versus open distal pancreatectomy: a meta‐analysis. Asian J Surg. 2012;35(1):1–8. 22726557 10.1016/j.asjsur.2012.04.001

[35] Xie K , Zhu YP , Xu XW , Chen K , Yan JF , Mou YP . Laparoscopic distal pancreatectomy is as safe and feasible as open procedure: a meta‐analysis. World J Gastroenterol. 2012;18(16):1959–67. 22563178 10.3748/wjg.v18.i16.1959 PMC3337573

[36] Pericleous S , Middleton N , McKay SC , Bowers KA , Hutchins RR . Systematic review and meta‐analysis of case‐matched studies comparing open and laparoscopic distal pancreatectomy: is it a safe procedure? Pancreas. 2012;41(7):993–1000. 22836858 10.1097/MPA.0b013e31824f3669

[37] Nakamura M , Nakashima H . Laparoscopic distal pancreatectomy and pancreatoduodenectomy: is it worthwhile? A meta‐analysis of laparoscopic pancreatectomy. J Hepatobiliary Pancreat Sci. 2013;20(4):421–8. 23224732 10.1007/s00534-012-0578-7

[38] Mehrabi A , Hafezi M , Arvin J , Esmaeilzadeh M , Garoussi C , Emami G , et al. A systematic review and meta‐analysis of laparoscopic versus open distal pancreatectomy for benign and malignant lesions of the pancreas: it’s time to randomize. Surgery. 2015;157(1):45–55. 25482464 10.1016/j.surg.2014.06.081

[39] Ricci C , Casadei R , Taffurelli G , Toscano F , Pacilio CA , Bogoni S . Laparoscopic versus open distal pancreatectomy for ductal adenocarcinoma: a systematic review and meta‐analysis. J Gastrointest Surg. 2015;19(4):770–81. 25560180 10.1007/s11605-014-2721-z

[40] Gavriilidis P , Roberts KJ , Sutcliffe RP . Laparoscopic versus open distal pancreatectomy for pancreatic adenocarcinoma: a systematic review and meta‐analysis. Acta Chir Belg. 2018;118(5):278–86. 29996721 10.1080/00015458.2018.1492212

[41] van Hilst J , Korrel M , de Rooij T , Lof S , Busch OR , Groot Koerkamp B , et al. Oncologic outcomes of minimally invasive versus open distal pancreatectomy for pancreatic ductal adenocarcinoma: a systematic review and meta‐analysis. Eur J Surg Oncol. 2019;45(5):719–27. 30579652 10.1016/j.ejso.2018.12.003

[42] Correa‐Gallego C , Dinkelspiel HE , Sulimanoff I , Fisher S , Viñuela EF , Kingham TP , et al. Minimally‐invasive vs open pancreaticoduodenectomy: Systematic review and meta‐analysis. J Am Coll Surg. 2014;218(1):129–39. 24275074 10.1016/j.jamcollsurg.2013.09.005

[43] Lei P , Wei B , Guo W , Wei H . Minimally invasive surgical approach compared with open pancreaticoduodenectomy: a systematic review and meta‐analysis on the feasibility and safety. Surg Laparosc Endosc Percutan Tech. 2014;24(4):296–305. 24743678 10.1097/SLE.0000000000000054

[44] Qin H , Qiu J , Zhao Y , Pan G , Zeng Y . Does minimally‐invasive pancreaticoduodenectomy have advantages over its open method? A meta‐analysis of retrospective studies. PLoS One. 2014;9(8):e104274. 25119463 10.1371/journal.pone.0104274 PMC4132100

[45] Pędziwiatr M , Małczak P , Pisarska M , Major P , Wysocki M , Stefura T , et al. Minimally invasive versus open pancreatoduodenectomy—systematic review and meta‐analysis. Langenbecks Arch Surg. 2017;402(5):841–51. 28488004 10.1007/s00423-017-1583-8 PMC5506213

[46] Chen K , Pan Y , Liu X‐L , Jiang G‐Y , Wu D , Maher H , Cai X‐J , et al. Minimally invasive pancreaticoduodenectomy for periampullary disease: a comprehensive review of literature and meta‐analysis of outcomes compared with open surgery. BMC Gastroenterol. 2017;17(1):120. 29169337 10.1186/s12876-017-0691-9 PMC5701376

